# Annual Report of the Physicians of the Marine Hospital, Quarantine, Staten Island, N. Y.

**Published:** 1850-07

**Authors:** 


*Annual Report of the Physicians of the Marine Hospital, Quarantine, Staten Island, N. Y.* Made to the Legislature March 26, 1850. By F. Campbell Stewart, M. D., Physician to the Hospital.

From this report we select as follows:

The greatest number of patients under treatment, at any one time since the first of July, and subsequent to the closing of the public stores, was less than three hundred and fifty; and the smallest number two hundred and twenty-eight.

| | | |
|---|---|---|
| The number of patients remaining in the several hospitals and public stores on the 23d of April, as ascertained by actual count, was | | 695. |
| Number admitted from 23d April to 31st of December, | | 2,520 |
| Whole number under treatment during the period indicated, - - - - - - - - . - | | 3,215 |
| Of these, there were discharged cured or relieved, | 2,369 | |
| Died, including 175 from cholera, - - - - | 503 | |
| Remaining in hospital Dec. 31st., - - - - | 343 | |
| | | 3,215 |

The per centage of deaths, including those from cholera, was about fifteen and a half per cent, and per centage, excluding the cholera cases and deaths, but including ship fever, small pox and all other diseases, was eleven and one-third per cent.

Of the whole number admitted into the institution, between the dates specified,

| | | |
|---|---|---|
| There were received from on board of vessels directly, | 1,010 | |
| The city and Ward's Island, (under authority from the board of health and Commissioners of Emigration, and for the most part, newly arrived emigrants,) - - | 1,478 | |
| Cases of emergency, cholera, small pox, &c, from Staten Island, chiefly emigrants, and sailors from the Seaman's Retreat, - - - - - - - - - | 32 | |
| | | 2,520 |

Excluding the numerous cases of extreme debility and exhaustion, and also such diseases as presented typhoid symptoms as an accompaniment only, there were received, from the 23d April to 31st December, 813 cases of genuine typhus fever, of which 112 died, giving a mortality of less than 14 per cent.—a result which should be entirely satisfactory to those who are familiar with the nature of this formidable disease.

Previous to the appearance of the cholera in New York city, many cases of that disease had occurred at the quarantine. Indeed, the Marine Hospital had never been entirely free from it since its first introduction by the packet ship "New York," in December, 1848.

The whole number of cases received into the institution, subsequent to the 23d of April, was 303, of which 175, or about 57¾ per cent., proved fatal. This is apparently a large mortality, but, in reality, not greater than the average in other public institutions, or in private practice; for it must be borne in mind that nearly all the cases were sent on shore from vessels arriving from sea, and the patients were often landed in a dying condition, and in so far advanced a stage of the disease, as to preclude the possibility of their deriving benefit from medical treatment. Among the deaths, too, are included some who were inmates of the institution prior to the 23d of April, while the admissions, with this disease, only included such as were received subsequent to that date.

After the cholera had ceased to prevail as an epidemic in the city of New York, numerous cases continued to be received into the Marine Hospital from vessels arriving from sea. The board of health discontinued their daily reports on the 5th of Septemcer, between which time and the 20th of November, 178 cases of cholera and cholerine were admitted from on board the following vessels, which had lost, in the aggregate, at sea, 348 of their passengers, most of whom are presumed to have died of the same disease.

The number of cases of small pox and varioloid received into the Marine hospital, from April 23d to December 31st, 1849, was 256; of which 22 only died, giving a mortality of less than nine per cent. for one of the most formidable and fatal diseases that medical men have to encounter. Several of the sick were affected with the most violent form of the confluent variety of small pox, and it is worthy of being noted, that a very large proportion of cases occurred among German emigrants, who are among the most cleanly and healthy of those who come to our shores, and who are subjected at home to strict sanitary regulations in regard to vaccination. A number of sailors are also included in the sum total of patients admitted with small pox and varioloid, and this class of citizens appears to be peculiarly liable to contract the disease.

